# In-Situ Monitoring of Directed Energy Deposition Laser Beam of Nickel-Based Superalloy via Built-in Optical Coaxial Camera

**DOI:** 10.3390/s25237348

**Published:** 2025-12-02

**Authors:** Rustam Paringer, Aleksandr Khaimovich, Vadim Pechenin, Andrey Balyakin

**Affiliations:** 1 Department of Technical Cybernetics, Samara National Research University, Moskovskoye Shosse 34, 443086 Samara, Russia; rusparinger@ssau.ru; 2 Department of Engine Production Technology, Samara National Research University, Moskovskoye Shosse 34, 443086 Samara, Russia; berill_samara@bk.ru; 3 Research Laboratory ‘Artificial Intelligence in Production Systems’, Samara National Research University, Moskovskoye Shosse 34, 443086 Samara, Russia; vadim.pechenin2015@gmail.com

**Keywords:** additive manufacturing, directed energy deposition, process monitoring, machine learning

## Abstract

This study presents the development and validation of an in situ monitoring method for the laser direct energy deposition (DED) process, utilizing an integrated optical camera (720 HD, 60 fps) to analyze melt pool imagery. The approach is grounded in an experimental framework employing Taguchi orthogonal arrays, which ensures a stable dataset by controlling process variability and enabling reliable extraction of relevant features. The monitoring system focuses on analyzing brightness distribution regions within the melt pool image, identified as specific clusters that reflect external process conditions. The method emphasizes precise segmentation of the melt pool area, combined with automatic detection and classification of cluster features associated with key process parameters—such as focus distance, the number of deposited layers, powder feed rate, and scanning speed. The main contribution of this work is demonstrating the effectiveness of using an optical camera for DED monitoring, based on an algorithm that processes a set of melt pool identification features through computer vision and machine learning techniques, including Random Forest and HistGradient Boosting, achieving classification accuracies exceeding 95%. By continuously tracking the evolution of these features within a closed-loop control system, the process can be maintained in a stable, defect-free state, effectively preventing the formation of common process defects.

## 1. Introduction

Data-driven process monitoring is becoming increasingly popular in the additive manufacturing process because it enables real-time verification of component quality. Qualifying additively manufactured parts in real time offers a considerable advantage by reducing the costs associated with traditional post-production inspection methods [1,2].

A great deal of research has been conducted into in situ quality monitoring. Broadly speaking, the main monitoring methods fall into three categories: surface and internal defect control methods, thermal analysis and melt pool and heat-affected zone parameter methods, and 3D surface micro-relief parameter methods. Detailed information on sensors and research methods can be found in reviews [3,4,5].

In this study, the main focus with regard to laser direct energy deposition (DED) technology is on optical measurement methods, since a built-in optical camera is used to take fragmentary images of the molten pool. The following technologies and sensors are used in optical systems:

(a) Visual Imaging with Optical digital camera/Photodiode for melt pool analysis [6,7,8,9,10,11,12,13,14,15,16], data collection for machine learning and defect detection [17,18,19,20], melt pool analysis and defect detection [21,22,23,24].
(b) Visual Imaging/High-fidelity Video with High frame-rate optical camera for melt pool analysis, powder consolidation [25,26,27,28,29,30,31,32,33,34,35].
(c) Thermography with IR Camera for melt pool analysis [28,36,37,38,39,40,41,42].
(d) Combine technologies Visual Imaging plus Thermography with Optical cameras and Pyrometer for Defect Detection [30,31,32,33], Visual Imaging plus Acoustic Emissions with Optical cameras and Acoustic sensors for part quality and defect detection [43,44].

Various process measuring devices, such as digital cameras [5,6,7], high-speed cameras [8,9,10], infrared cameras [11,12,13], acoustic sensors [14,15,16,17] and photodiodes [18,19,20,21] are usually the first to be implemented around the process zone in order to collect process signatures.

While incorporating suitable optics with IR and high-speed imaging techniques enables the retrieval of high-resolution spatial information from the melt pool surface, issues arise with emissivity calibration [36,37,38,39]. Additionally, it should be emphasised that this approach is not cost-effective in terms of hardware management, data management and treatment. Furthermore, the optics must be mounted co-axially with the laser head to improve visualization of the melt pool, requiring alterations to existing industrial machines.

Experiments focusing on powder flow measurements and the mechanisms of powder delivery in Directed Energy Deposition (DED) processes have been instrumental in optimising powder flow rates and spatial powder distribution distances [40,41]. A range of microstructural changes can occur within the melt pool, including the melting and vaporisation of powders and the substrate, powder movement and ejection, spattering, the solidification of molten materials and powders, and alterations to the vapour depression zone. All of these changes can contribute to defect formation [28,30,31,32,33,34,35,36,37,38,39,40,41,42,43,44,45].

The main objects of monitoring are structural defects, which in turn depend on processing parameters (factors).

Elevated temperatures, rapid thermal cycling and significant temperature gradients can affect the energy density of the process, potentially causing defects in the manufactured parts [46,47]. Consequently, defect formation is strongly linked to the temperature gradients present during processing. This is supported by extensive research into laser welding and laser cladding, which share similarities with LPBF in theory and methodology [48,49,50].

LPBF parameters, such as laser power, powder composition, scanning speed, layer thickness, scanning strategy, chamber environment and particle size, directly affect part quality [51,52]. Sub-optimal laser settings can result in defects such as balling, cracking, delamination, lack of fusion (LoF) and keyhole porosity, which reduce density and weaken mechanical properties. High scanning speeds increase melt pool instability, causing spheroidisation (balling) to minimise free energy [53,54], while insufficient laser power reduces thermal energy, impairing wettability and fluidity and also leading to balling [55,56].

Balling increases surface roughness and pore formation. LoF defects result from inadequate energy, which causes incomplete laser track overlaps and traps unmelted powder, creating permanent pores [57,58]. In contrast to balling and LoF, keyhole porosity occurs when high thermal energy density creates deeper melt pools and vapour channels [59]. The collapsing vapour cavity traps metal vapour and shielding gases, forming spherical pores [29]. Keyhole pore formation under different laser scanning speeds demonstrates that keyhole pores are sensitive to manufacturing parameters [60].

**Figure 1:**
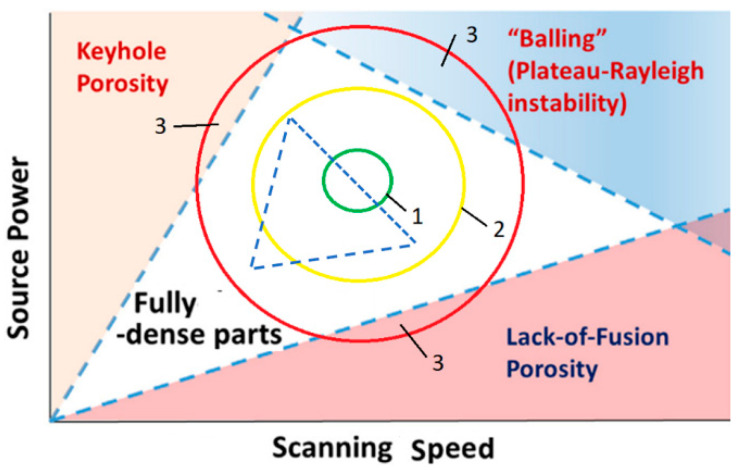
Illustration of processing parameter influence on porosity: 1—optimum zone, 2—permissible zone, 3—dangerous zone. Reconstructed from [61]. The small blue triangle indicates the possible area of fully dense parts when external conditions change, for example, the temperature of the fused layer.

Figure 1 illustrates the influence of processing parameters on porosity: keyhole porosity appears in the high-power/low-velocity regime, whereas lack-of-fusion porosity appears in the low-power/high-velocity regions of the processing parameter space [61].

Advancements in sensor technology, coupled with the ability to extract valuable information from sensor signatures during manufacturing using machine learning (ML) algorithms, have become crucial for developing effective, data-driven monitoring systems. Conventional ML techniques, such as K-Nearest Neighbour (KNN) and Decision Trees, have been used with optical and thermal sensor data to evaluate part quality [62]. Khanzadeh et al. [63] applied multilinear principal component analysis (MPCA) to thermal maps for process monitoring. Gobert et al. [64] trained a linear support vector machine (SVM) on visual features derived from images of the build layer, while features obtained via singular value decomposition (SVD) from photodiode sensors were used within a Gaussian mixture model (GMM) to evaluate quality [65]. Acoustic signals analysed using SVM, Random Forest (RF) and logistic regression classifiers have also proven effective in identifying processing regimes [66,67]. However, these approaches rely on manual feature extraction, necessitating human expertise. In contrast, deep learning techniques can process raw sensor data directly with minimal preprocessing, presenting a promising alternative for monitoring LPBF processes [68,69].

Machine learning (ML), particularly deep learning (DL), is widely used to analyse in situ sensing data in manufacturing, correlating signals to quality-related features such as melt flow [50,70], melt pool [71,72], and porosity defects [73,74], among others [68,75,76]. ML techniques can effectively uncover hidden knowledge and complex relationships within digital manufacturing systems, learning from reliable training datasets to make decisions without the need for explicit programming [77]. However, balanced datasets are essential for optimal model performance, which can be challenging in real-world applications due to data imbalance, where one class (the majority) exceeds the others (the minority). As demonstrated by Scime et al. [78], the classification accuracy of minority classes is notably lower, and anomaly samples are often misclassified as normal. This imbalance hinders the model’s ability to detect defects, since acceptable conditions are more prevalent than defective ones in practice, resulting in datasets that are biased towards normal conditions. For this reason, Li et al. propose an imbalanced data generation and fusion approach for in situ quality monitoring in LPBF [79]. They capture layer-wise images, acoustic signals and photodiode signals with a multi-sensor system and then create three intentionally imbalanced datasets. A generative adversarial network-based data generation model (GAN-based model) generates minority class samples, and a deep learning (DL)-based fusion method combines the augmented datasets. The results demonstrate that high-quality generated samples substantially enhance model performance.

Machine learning algorithms can address many challenges in process monitoring by enabling the quick extraction and analysis of features, which is suitable for real-time LPBF applications. However, most current methods rely on passive supervised learning, which is time-consuming and costly in terms of training and testing. Exploring unsupervised and reinforcement learning approaches could greatly improve the quality control of real-time LPBF processes [79].

This study proposes a new approach to creating a dataset for monitoring L-DED processes, based on the fact that defects are formed when fusion process parameters shift into a dangerous range due to external disturbances (Figure 1).

Let us illustrate this with the example of a change in focal length. Defocusing can occur due to the accumulation of layer-by-layer errors as a result of powder consumption not matching the theoretical increase in deposited volume.

**Figure 2:**
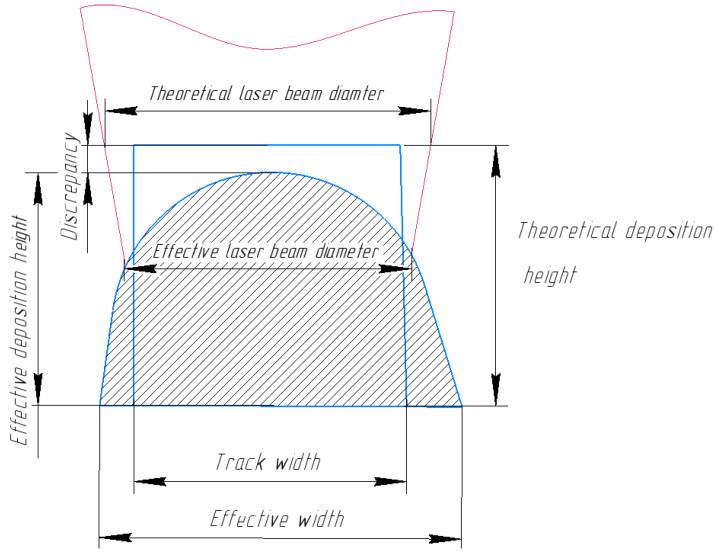
Geometric attributes measured from the 3D scan.

The actual profile of the deposited weld bead differs from the theoretical profile, which is based on the volume of powder supplied to the melt pool. This discrepancy (see Figure 2) increases with each subsequent layer as the total deposition height grows. Consequently, defocusing occurs, and the actual radius of the laser beam differs from the theoretical radius. This deviation can occur in either direction, making the radius larger or smaller. Consequently, the temperature field in the melt pool and the temperature of the keyhole surface are affected.

By using the 2D moving heat source model [29], the keyhole surface temperature is approximated by the melt pool peak temperature,

(1) $$T=\frac{\sqrt{2}\beta Ir}{k\sqrt{\pi }}\tan^{-1}\sqrt{\frac{2a}{v_{l}r}}$$

where *I* is the laser intensity (approximated as $I=P/(\pi r^{2})\;$), k=ρ·c·a is the liquidus thermal conductivity, r is the laser beam radius. β is the laser absorptivity, a is the liquid thermal diffusivity, and P is laser power. Keyhole porosity depends on several factors, including the peak temperature of the melt pool. According to Equation (1), this temperature is a function of the actual laser beam radius r, and the velocity of the liquid in the melt pool is $v_{l}$. The latter is conventionally equated to the scanning speed and is proportional to the laser power [80]. Therefore, controlling the focal length enables one of the factors influencing defect formation to be controlled. The same applies to other technological parameters, primarily radiation power and scan speed. Key factors influencing the occurrence of defects include the temperature field in the melt bath and HAZ, local temperature fluctuations, and temperature changes over time.

During metal deposition, it should be noted that the heat balance conditions for each layer change as the total deposition height increases due to the accumulation of thermal energy in the total deposited volume. This results in a gradual shift in the boundaries of the defect regions within the technological parameter coordinates (see Figure 1). Consequently, the regions of optimal parameter 1 and acceptable parameter 2 (Figure 1) will also shift from layer to layer. Controlling the technological parameters (radiation power, scanning speed and the technological pause necessary to equalise the heat balance in each layer) ensures that these values do not exceed the permissible range 2 (Figure 1).

The experimental objective was to predict process parameters such as welding speed, effective focusing distance, internal powder flow rate, and the number of the deposited layers. Since each of these parameters takes on a limited set of values, the prediction task was transformed into a classification problem, where each unique value of the target parameter corresponds to a specific class. For each target parameter, a separate classifier was trained.

## 2. Materials and Methods

Let us formulate the control problem as follows: The target state of the DED system is an absence of defects. This state is determined by a vector of target parameters ${\left\{P,v,f\right\}}_{0}$ for laser power, scanning speed and focus distance, provided that the technological parameters affecting the melt pool and crystallisation process remain unchanged.

Let’s assume that it is possible to create a mapping between the technological parameters {P,v,f} by preprocessing the images of the melt pool obtained from the sensor and transforming them into a certain set of *k = 1…K* features $\left\{x_{k}\right\}$, which identify these images.

Target state ${\left\{P,v,f\right\}}_{0}$ corresponds to a vector of features, such that the following dependencies are satisfied:

(2) $${\left\{P,v,f\right\}}_{0}=\left\{F_{P}^{0}({\left\{x_{k}\right\}}_{0}),F_{v}^{0}({\left\{x_{k}\right\}}_{0}),F_{f}^{0}({\left\{x_{k}\right\}}_{0})\right\}$$

where $F^{i}$ some fuzzy matching functions are defined on a set of permissible parameters $R^{i}$

$$\left\{F_{P}^{i}({\left\{x_{k}\right\}}_{i}),F_{v}^{i}({\left\{x_{k}\right\}}_{i}),F_{f}^{i}({\left\{x_{k}\right\}}_{i})\right\}\subset R^{i}$$

For example, the set of parameters $R^{0}$ corresponds to area 1, and the set of parameters $R^{1}$ corresponds to area 2 (Figure 1). In this case, it is necessary to solve the following control problem:

(3) $${\left\{x_{k}\right\}}_{i}\underset{R^{i}\to R^{0}}{\to }{\left\{x_{k}\right\}}_{0}$$

i.e., by influencing the technological parameters ${\left\{P,v,f\right\}}_{i}$, ensure that condition (3) is met.

In our case, such a sensor is the optical camera of the DED device.

The permissible range of parameter R is selected in such a way that the shift in this region for any i-th layer $R^{i}=R^{0}+\delta R$ does not cause the appearance of defects.

This problem can be solved by robust design of experiments based on Taguchi methods [81,82] when varying factors ${\left\{P,v,f\right\}}_{i}$ within specified limits δR.

As a result, instead of the need to generate a DataSet for implementing control algorithms based on ML with samples containing defect signatures—which, as previously mentioned, is labor-intensive and material-specific—it becomes possible to perform in situ monitoring of the DED process. This is achieved by collecting data from sensors and transforming it into signature parameters, thereby ensuring the control law (3).

Summarizing and moving towards the main goal of this paper—namely, in situ monitoring of the DED process using the optical camera—the following problems should be solved:

(a) Identification of a sufficient set of signature parameters $\left\{x_{k}\right\}$, obtained from the camera mounted coaxially with the laser head, is necessary for the identification of the melt pool state.
(b) Solution of a classification problem, using ML mechanisms to develop dependencies (3) for variables P and v with an acceptable accuracy of no less than 95%, aimed at process regulation.

A separately defined task involves deriving regression equations that relate the laser beam spot radius r to the signature parameters $\left\{x_{k}\right\}$, $r=r_{fuzzy}(\{x_{k}\})$.

**Figure 3:**
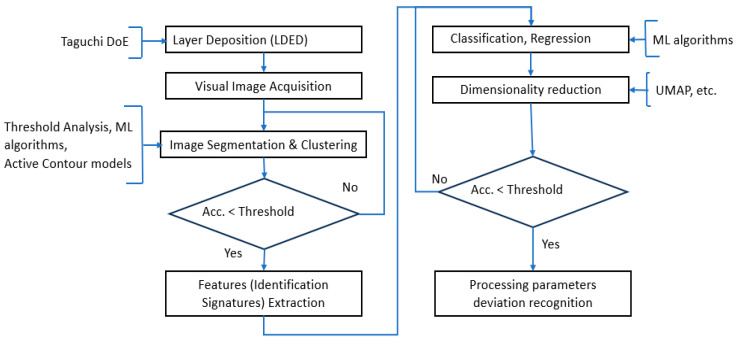
The overall research logic.

The overall research logic is presented in Figure 3.

Thus, the main goal is to determine whether it is possible to predict the welding mode parameters based on features obtained from the formed segmentation masks. This will allow assessing the effectiveness of the technology for contactless real-time monitoring of DED.

**Figure 4:**
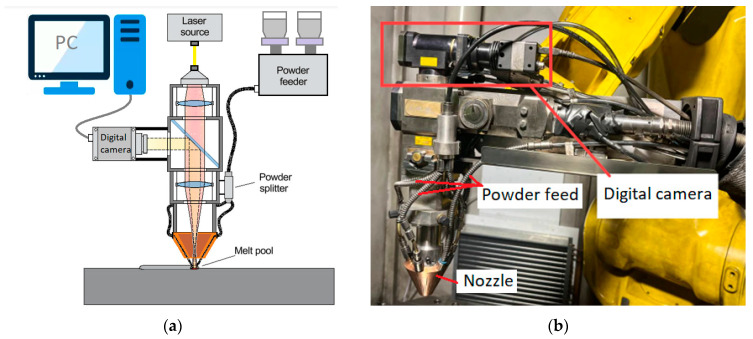
Schematic representation (**a**) of the DED in situ monitoring system with the built-in optical camera (**b**).

All experiments were conducted using ILIST (ILIST, Saint Petersburg, Russia; Nd:YAG, 2000 W, continuous-wave laser) with a laser beam movement system, powder feeding system, coaxial nozzle based on the Fanuc 6-axis robot, and a 2-axis rotary platform. Melt pool imaging was performed with the built-in P30-007443-HD Camera (True 720p HD output @ 60 fps), which was supplied together with the IPG D30 welding head (Figure 4).

The process parameters for deposition of specimens in the form of rectangular parallelepipeds with dimensions (L × W × H) of 50 × 10 × 10 mm made from high-temperature corrosion-resistant alloy Cr32Ni50W4.5Mo2.5TiAl powder (Table 1) for the orthogonal L9:4 Taguchi experimental plan for 3 factors are presented in Table 2 and Table 3. The diagram of image registration of the melt pool for creating the DataSet for training and validation of the monitoring system is shown in Table 4.

For the experiments, the built-in system recorded video recordings of the metal deposition processes under various process modes. The video files were saved in the format “X Y Z.mp4”, where

- X—the number of the process mode (from 1 to 9);
- Y—the layer number by height of the specimen {2, 4, 5, 7, 12};
- Z—the sequential number of the track within the layer (from 1 to 5).

For each video, segmentation masks were generated to highlight important regions: the exposure area (background inside the powder feed nozzle), the laser spot, sparking region around the laser spot, the high-temperature region, and other areas. Based on these masks, new parameters (features) were calculated and recorded in CSV files with the same names as the video recordings. A description of these new parameters is provided below in Table 5.

The features are grouped in triplets: the area of the region of interest and its center coordinates (X and Y). The calculation of these parameters was performed based on segmented images using contour analysis methods from the OpenCV library (https://opencv.org (accessed on 26 November 2025). This dataset serves as the basis for analyzing and predicting the process state (addressing classification and regression tasks) based on the extracted features.

Across all experiments, the dataset was divided into training and testing subsets in an 80% to 20% ratio. The split was stratified to preserve the class proportions.

For building classifiers, models from the scikit-learn library were used. To evaluate the quality of each model, the following metrics were calculated:

- Accuracy is the proportion of correct predictions.
  (4) $$Accuracy=\frac{TP+TN}{TP+TN+FP+FN},$$
  where
  TP is the number of true positives;
  TN is the number of true negatives;
  FP is the number of false positives;
  FN is the number of false negatives.
- Precision is the proportion of correct predictions of a given class among all predictions of this class.
  (5) $$Precision=\frac{TP}{TP+FP}$$
- Recall is the proportion of correct predictions of this class among all actual observations of this class.
  (6) $$Recall=\frac{TP}{TP+FN}.$$
- F1-score is a harmonic mean of precision and recall.
  (7) $$F1=2\cdot \frac{Precision\cdot Recall}{Precision+Recall}$$

For each model, the averages of these metrics and their standard deviations were calculated. The main metric for comparison is the F1 measure, which reflects the balance between precision and recall.

The following characteristics were used to assess the quality of the regression models:

- The coefficient of determination $R^{2}$, which describes the relationship between actual and predicted values:

(8) $$R^{2}=1-\frac{\sum_{i=1}^{N}{\left(y_{i}-{\hat{y}}_{i}\right)}^{2}}{\sum_{i=1}^{N}{\left(y_{i}-\bar{y}\right)}^{2}},$$

$$\bar{y}=\frac{1}{N}\sum_{i=1}^{N}y_{i}$$

- The Mean Absolute Error (*MAE*)—an estimate of the difference between actual and predicted values:

(9) $$MAE=\frac{1}{N}\sum_{i=1}^{N}\left|y_{i}-{\hat{y}}_{i}\right|$$

where $y_{i}$ is actual value, ${\hat{y}}_{i}$ is predicted value i=1…N, and N is the total number of values.

The following material was used to clarify the analysis tools in the form of graphs: Confusion matrices, along with bar graphs, were employed to visually represent classification performance. A confusion matrix evaluates a classification model by comparing its predicted outcomes to the observed outcomes in the dataset. It displays the number of correct predictions (true positives and true negatives)—located on the main diagonal from the top-left to the bottom-right—and incorrect predictions (false positives and false negatives)—found in the off-diagonal elements. 1D and 2D bar graphs were utilized to illustrate the values of various quality metrics. The 2D bar graphs were constructed as arrays of colored rectangular cells, where the color intensity of each cell reflects the value of the quality metric at the corresponding coordinate (X, Y). To assess regression performance, 2D plots of classification quality metrics and scatter plots were employed.

## 3. Results

### 3.1. Results

**Figure 5:**
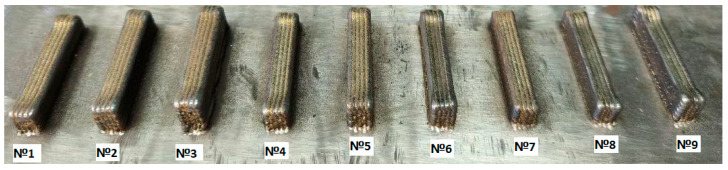
Appearance of specimens obtained according to the experiment design for 3 factors.

Photos of the fabricated specimens, in accordance with the experimental plan (Table 2), are presented in Figure 5.

**Figure 6:**
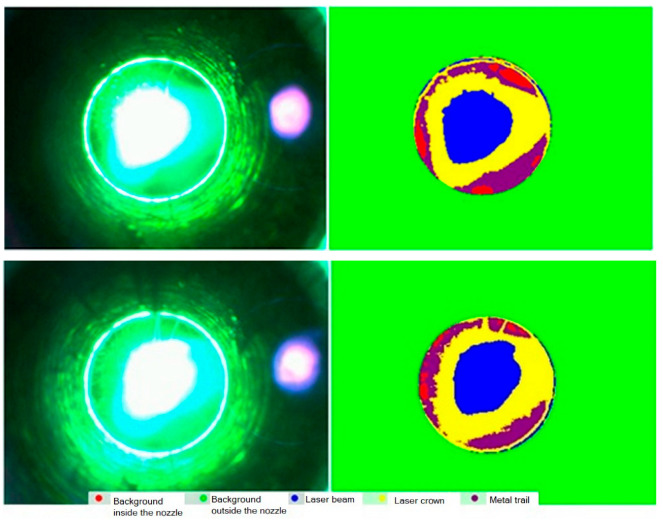
Some results of segmentation based on brightness thresholding and subsequent correction using PSPNet based on PyTorch 2.9.1.

Examples of segmentation of the recorded images of the molten pool based on brightness threshold analysis with subsequent correction of brightness front distribution using the PSPNet neural network framework (https://pytorch.org (accessed on 26 November 2025) for Python 3.14.0. machine learning are shown in Figure 6. Threshold processing is used to extract the background of the melt pool image area. The background area is not of interest but can induce false positive results in the neural network’s operation. Therefore, the region of interest (ROI), which is fed into the neural network for segmentation, contains only the central part of the image (bounded by the circle of the nozzle contour—see Figure 6), excluding background pixels. Pixels within the ROI are normalized based on brightness and scaled to a mean value of 128 with a variance of 64. The shape of the ROI is extended to a square by padding with zeros, and the resulting normalized image is then input to PSPNet. The contours of the nozzle were determined based on the analysis of the first several frames using threshold processing.

For each frame of the recorded video, regions of interest were identified based on the segmentation mask: laser spot, sparking region around the laser spot, the high-temperature region, and background. In each region, the largest contour (by area) was initially located using the cv2.findContours function from the OpenCV framework, which detects all possible contours in the image. Then, key parameters such as area S and centroid coordinates (*X* and *Y*) were calculated for the largest contour using moments via cv2.moments:

(10) $$S=m_{00},X=\frac{m_{10}}{S},Y=\frac{m_{01}}{S},$$

where $m_{10}$ and $m_{01}$ are the first-order moments, and $m_{00}$ is the zero-order moment representing the contour area.

These parameters were computed for each frame using the contours of segmented images. To determine the number of identifying features of the segmented regions, their normalization was performed relative to the geometric characteristics of the entire region of interest (area and nozzle center coordinates).

The resulting data were aggregated into a Dataset according to the variation levels of the parameters (Table 6). The dataset contains a different number of frames from various layers: 2—1314, 4—1339, 5—1377, 7—1320, 12—1315. Layers 2, 7, and 12 are used for classification tasks, as these layers did not involve focus distance variations. These layers contain approximately the same amount of data, with the total specimen size being 3949 elements. Layers 4 and 5, with a total of 2716 elements, were used for experimental studies within the regression task, as focus distance variations on these layers were implemented according to the data in Table 4.

### 3.2. Data Analysis

For the analysis of the quality of the identifying features, their informativeness was evaluated based on their data separating ability using the metric of classification performance when applying a feature reduction method, UMAP (Uniform Manifold Approximation and Projection) [83]. A comparison was conducted between the classification results when the classifier was trained on a set of synthetic parameters of reduced dimensionality obtained by the UMAP method from an original set of 9 identifying features.

The analysis was performed in an 8-dimensional space of synthetic parameters (95% explained variance) and a 3-dimensional space of synthetic parameters. The classification quality was assessed using the F1-score on a dataset of 3949 segmented image features, each obtained from individual specimens produced under different technological regimes specified in Table 2 and Table 3. The analysis utilized the Random Forest Classifier.

**Figure 7:**
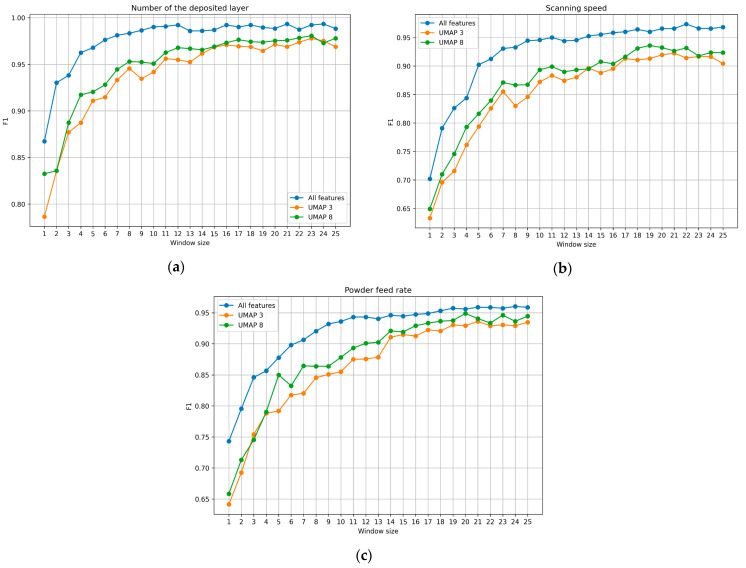
Dependence of F1 on the window size and the set of features (**a**)—number of the deposited layer, (**b**)—scanning speed, (**c**)—powder feed rate.

Within each window, data were smoothed using a moving average to reduce the influence of noise. The window size varied from 1 (no smoothing) up to 25 frames. Figure 7 shows the dependence of the classification quality metrics, specifically the F1-score, on the laser speed for input parameter sets of 9 features of the region of interest, 8 synthetic parameters, and 3 synthetic parameters derived from the original features. The classification results based on the layer number and powder consumption showed similar patterns, and the corresponding results are presented below.

**Figure 8:**
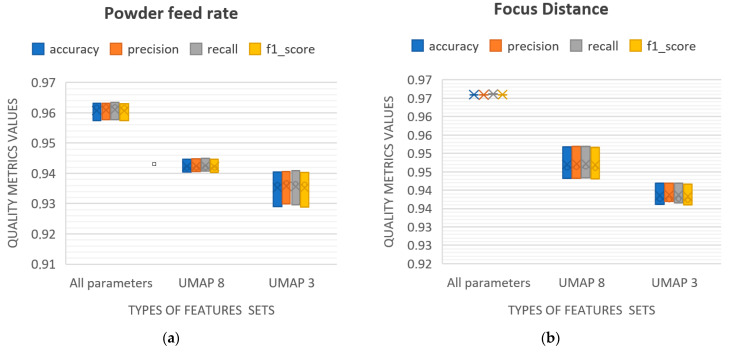

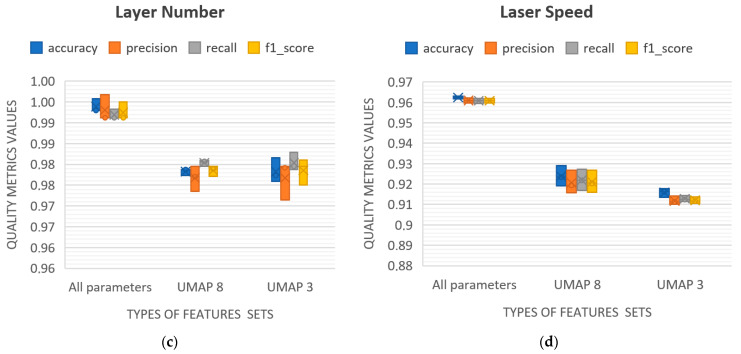
Box plots of averaged (within the size of 20…25 frames of the sliding window) classification quality metrics aggregated by sets of input parameters containing 3 types of features sets: all parameters—9 identifying features, UMAP8—8, UMAP—3 synthetic parameters (**a**)—powder feed rate, (**b**)—focus distance, (**c**)— number of the deposited layer, (**d**)—scanning speed.

As can be seen from the graphs in Figure 7, when the size of the moving window is within the range of 20 to 25 frames, the stabilization of classification quality is achieved regardless of the size of the input feature set. To analyze the impact of informational content and the number of identifying features on clustering quality, box plot diagrams of the classification quality metrics are presented in Figure 8. These plots allow for the evaluation of the variability in the classification quality metrics for the compared sets of identifying features as the classification process stabilizes using the sliding window method. The diagrams aggregate results based on input parameter sets containing, respectively, 9 identifying features, as well as 8 and 3 synthetic parameters. The analysis of the plots (Figure 8) shows that a reduction in the number of identifying features, as well as the formation of synthetic parameters from them—which, to varying degrees, affects the degrees of freedom of the data—has a significant impact on classification accuracy. This indicates the informational importance of each identifying feature from the original set of input parameters. Therefore, it can be reasonably concluded that the chosen set of identifying features is valid. The optimal size of the moving averaging window is 23 frames, based on the maximum sum of metric values across all three feature sets used.

### 3.3. Classification

An analysis was conducted to investigate how the accuracy of the prediction model depends on data aggregation from subsamples (folds), taking into account the layer number, scanning speed and powder feed rate. To obtain a more reliable assessment of the model’s real-world performance, 5-fold cross-validation was performed on the original dataset. Experiments were carried out using the Histogram-based Gradient Boosting Classification Tree model from the scikit-learn software package, with a maximum tree depth of 3. Data preprocessing involved averaging over a sliding window of 23 frames for each file. Layers 2, 7, and 12 and all tracks (1–5) were used for the experiment, since no focus distance variation was applied to these layers. The size of the entire dataset was 3949 elements.

(a) Number of deposited layers

**Figure 9:**
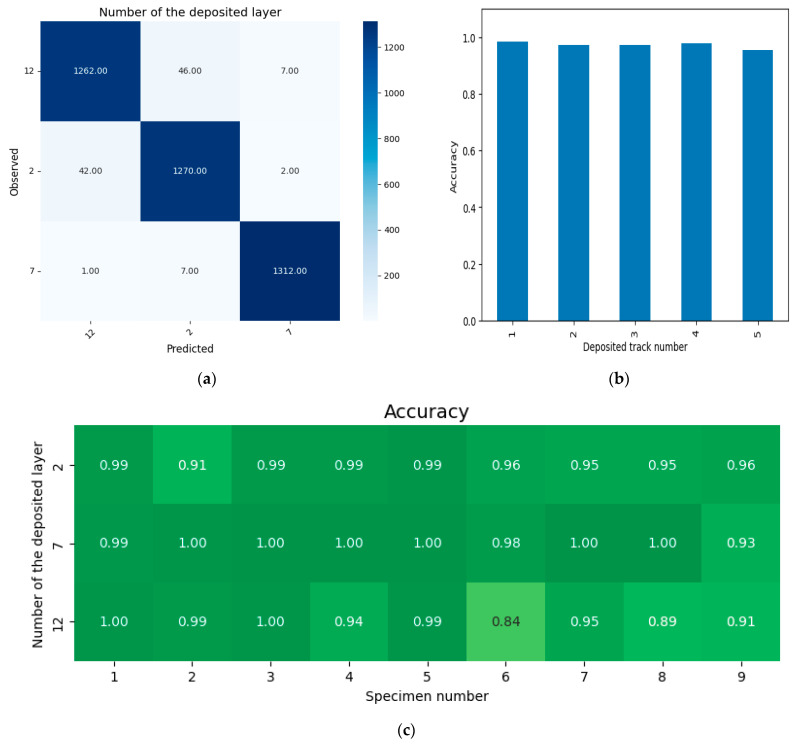
Classification results by the sequential number of the deposited layer: (**a**)—confusion matrix; (**b**)—average accuracy depending on the track number; (**c**)—accuracy depending on the layer number and process mode.

The classification dataset consisted of three classes labeled as 2, 7, and 12, corresponding to the actual number of deposited layers. The distribution of objects across classes was as follows: 2–1314, 7–1320, and 12–1315. The training set comprised 80% of the dataset, selected using a stratified sampling approach, with the following distribution: 2–1051, 7–1056, and 12–1052. The distribution of the test set was: 2–263, 7–264, and 12–263. The 5-fold cross-validation results for predicting the number of deposited layers, across variations in deposited track numbers and technological modes, are shown in Figure 9. The average accuracy for the entire dataset is 0.973. Analyzing the predicted versus true value confusion matrix (Figure 9a) demonstrates good stability in predicting the number of deposited layers across nearly all layers. The accuracy metric values for the middle layers (in Figure 9c, this is layer 7) are slightly higher than for the bottom and top layers—layers 2 and 12, respectively. This is because the specimen being built up accumulates a sufficient amount of heat in the middle layer, leading to poorer thermal conductivity; as each new track is deposited, the specific localization of heat allows for more precise determination of the number of deposited layers. For the upper layer, the relative increase in heat input becomes less significant, resulting in a slight decrease in accuracy. This pattern is characteristic of all technological modes (Figure 9c). It should also be noted that the accuracy of determining the deposited layer does not depend on the deposition track (Figure 9b).

(b) Scanning speed

**Figure 10:**
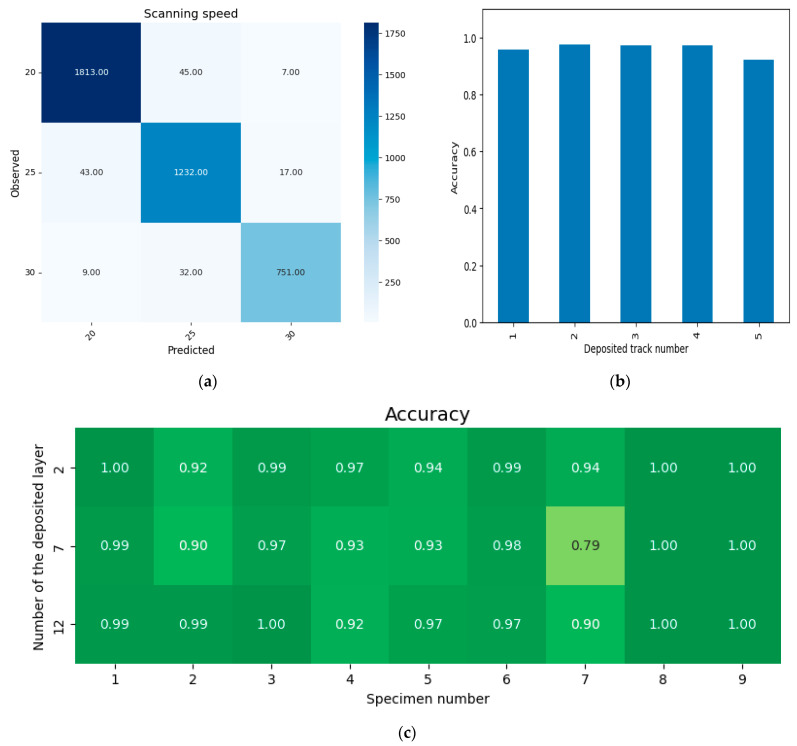
Classification results based on scanning speed: (**a**)—confusion matrix, (**b**)—average accuracy depending on the track number, (**c**)—accuracy depending on the layer number and process mode.

The classification dataset consisted of three classes labeled as 20, 25, and 30, corresponding to the specified scanning speed values. The distribution of objects across classes was as follows: 20–1865, 25–1292, and 30–792. The training set comprised 80% of the dataset, selected using a stratified sampling approach, with the following distribution: 20–1492, 25–1033, and 30–634. The distribution of the test set was: 20–373, 25–259, and 30–158. The 5-fold cross-validation results for predicting the scanning speed, across variations in deposited track numbers, layer numbers and technological modes, are shown in Figure 10. The average accuracy across the entire dataset is 0.961. The analysis of the confusion matrix (Figure 10a) shows that, as the scanning speed decreases, the classification accuracy increases. This phenomenon is associated with the fact that, at higher speeds, the boundaries of regions of interest become blurred, i.e., the laser spot, the sparking region around the laser spot, and the high-temperature region. As a result, the set of identifying features (Table 5) has a reduced discriminative capability for the classification task. It should be noted that this characteristic is observed almost equally across all process modes (Figure 10c) and for all deposition tracks (Figure 10b).

(c) Powder feed rate

**Figure 11:**
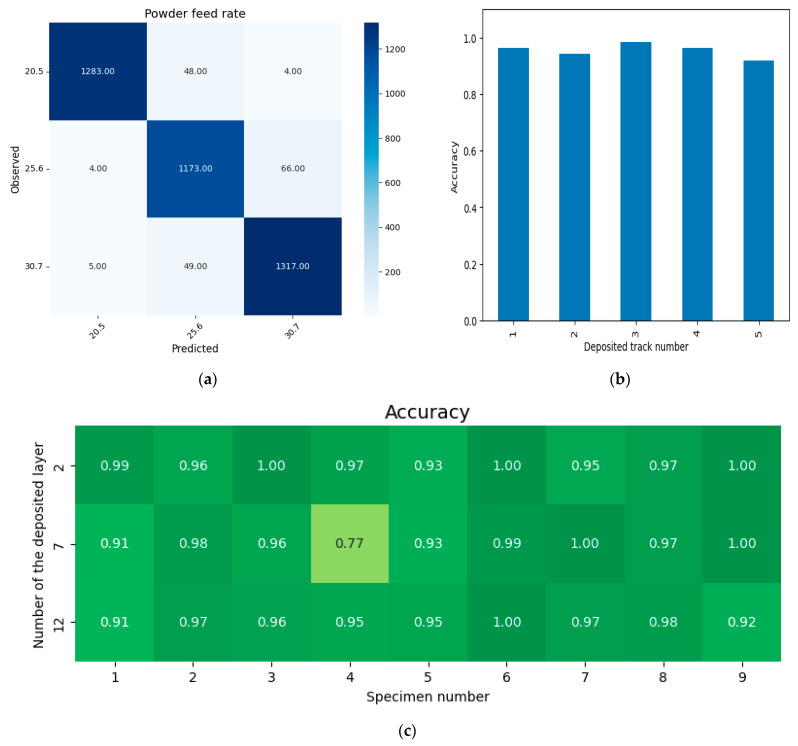
Classification results based on powder consumption: (**a**)—confusion matrix, (**b**)—average accuracy depending on the track number, (**c**)—accuracy depending on the layer number and process mode.

The classification dataset consisted of three classes labeled as 20.5, 25.6, and 30.7, corresponding to the specified powder feed rate values. The distribution of objects across classes was as follows: 20.5–1335, 25.6–1243, and 30.7–1371. The training set comprised 80% of the dataset, selected using a stratified sampling approach, with the following distribution: 20.5–1068, 25.6–994, and 30.7–1097. The distribution of the test set was: 20.5–267, 25.6–249, and 30.7–274. The 5-fold cross-validation results for predicting the powder feed rate, across variations in deposited track numbers, layer numbers and technological modes, are shown in Figure 11. The average accuracy across the entire dataset is 0.955. The analysis of the confusion matrix (Figure 11a) shows stable detection of powder feed rate across all process modes (Figure 11c) and for all deposition tracks (Figure 11b). However, when deviations of the powder feed rate from the nominal value of 25.6 g/min occur, the prediction accuracy slightly increases (Figure 11a—row 25.6 vs. row 20.5 and row 25.6 vs. row 30.7). This is explained by the fact that the error in deviations of powder feed rate from the nominal mode accumulates from layer to layer, which enhances the discriminative capacity of the set of identifying features and improves classification quality. It should be noted that powder feed rate is a key parameter for controlling the L-DED process, as deviations from the required powder feed rate, as previously noted, influence the actual focus distance. Therefore, a more precise method for analyzing these deviations would involve solving a regression task for the actual focus distance.

### 3.4. Regression

Experiments were conducted using regression models: Extra-Trees Regressor (ETR) and Gaussian Process Regression (GPR) from the sklearn software package. The data preprocessing involved averaging over a sliding window of 23 frames for each file. The experiment utilized layers 4 and 5 across all tracks (1–5), as these layers involved variation in the focus distance. The total sample size was 2716 elements. The validation set comprised 20% of the available data and was used to obtain estimates of regression quality.

For data analysis and solving the regression task, which aims to establish a relationship between the focus distance and process parameters, the importance (informational significance) of the identifying features was preliminarily assessed. During this analysis, a non-empty set of different combinations of features was generated through a brute-force approach, and the focus distance was predicted based on each set. The criterion for the optimal combination was the lowest Mean Absolute Error (*MAE*) value. The results of this analysis are presented in Table 7.

**Figure 12:**
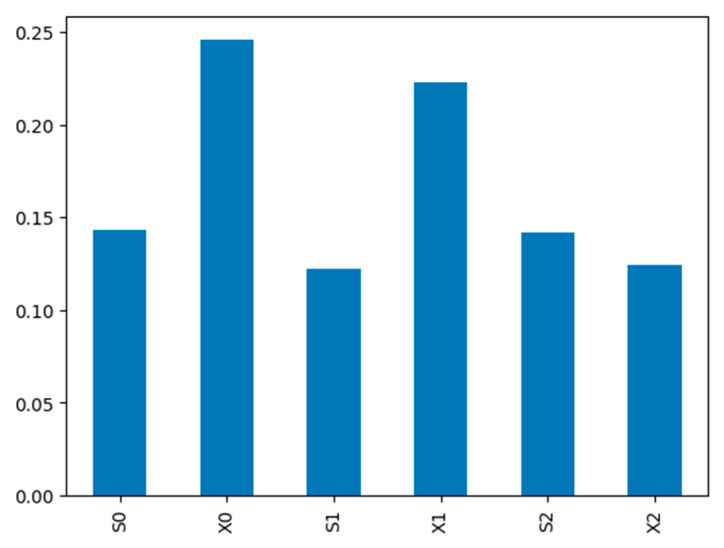
Feature importance weights from the Extra-Trees regressors.

As a result of the analysis, it was established that accuracy is achieved when using all features of area S and horizontal displacement X. Adding vertical displacement Y features does not lead to a significant reduction in error, which is consistent with the characteristics of the imaging process (movement primarily occurred in the horizontal direction of the camera’s field of view). Consequently, in the regression task, features $S_{0},\;X_{0},\;S_{1},\;X_{1},S_{2},\;X_{2}$, were used further. The weights associated with each feature, determined by the Extra-Trees regressor model, are presented in Figure 12.

**Figure 13:**
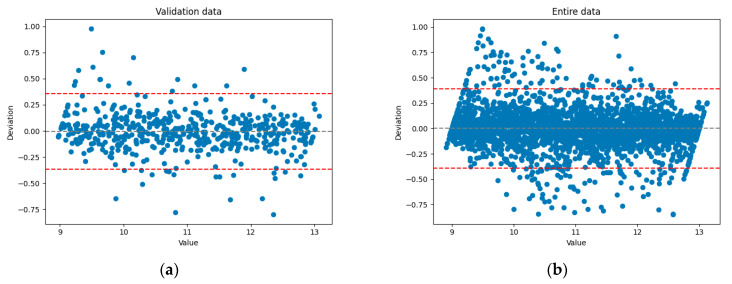
Bland–Altman [84] plots for the optimal feature combination. (**a**)—validation data, (**b**)—entire data.

The best results, with a coefficient of determination (R^2^) of 0.96, were achieved using a Gaussian process regression model. Bland–Altman plots illustrating the deviations of predicted values for the validation set and the entire dataset are shown in Figure 13a,b where the 2σ limits are indicated by red dashed lines.

**Figure 14:**
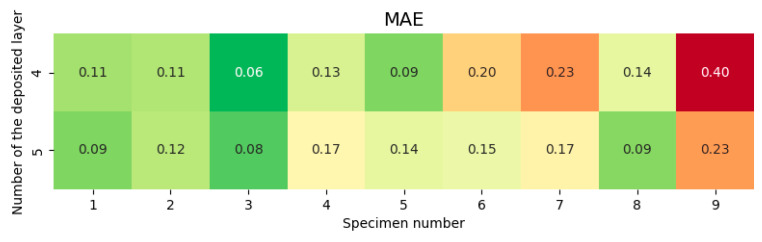
*MAE* dependence on technological mode (specimen number) and layer index.

**Figure 15:**
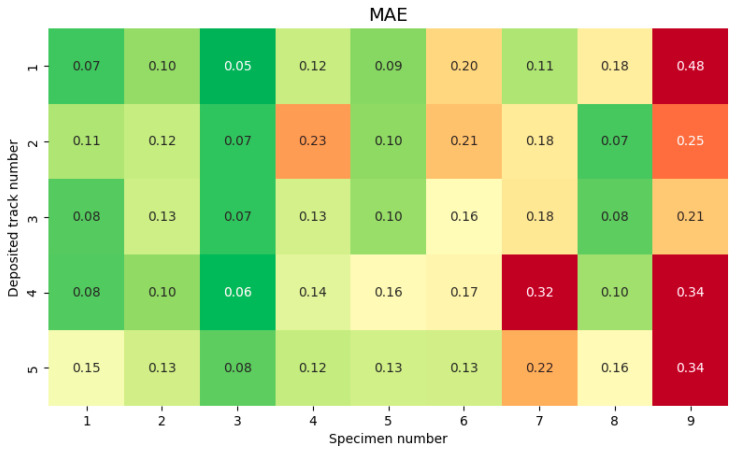
*MAE* dependence on technological mode (specimen number) and deposited track number.

The dependence of *MAE* on the technological mode (specimen number), layer number, and deposited track number is shown in Figure 14 and Figure 15.

## 4. Discussion

The analysis of the research results led to the following conclusions:

The application of robust experimental design and orthogonal Taguchi plans for image segmentation of the region of interest (monitoring the melt pool via built-in camera of the L-DED system) enabled the following tasks:

a Efficiently creating a dataset with video specimens featuring controlled variability of the melting process parameters.
b Improving the segmentation accuracy of images of the melt pool region of interest captured by the DED system’s camera, reducing the influence of factors causing image variations.

Controlled variability, based on experimental planning methods, allows variation in process parameters (powder feed rate, laser power, scanning speed) within specified levels that reflect possible real-world variations. The most significant variations influencing the results are related to practical deviations, primarily:

- Discrepancies between the calculated and actual powder feed rate, affecting focus distance and scanning speed.
- Variation in scanning speed, linked to the dynamics of DED-system acceleration and deceleration during changes in deposition direction.

Laser power is correlated with scanning speed via the linear energy density (P/V), which is necessary for stable DED operation (see Figure 1). Since the variations in power within the study were within manufacturer-recommended values, these were not explicitly varied.

It’s important to note that segmentation with neural networks is sensitive to shooting conditions. Adapting the segmentation algorithm to different camera settings, as well as ensuring reliable performance across different materials, is challenging. Therefore, for practical implementation, it is necessary to develop an algorithm to stably extract regions of interest based on image brightness in segmented images of the melt pool. The planned implementation will utilize standard computer vision tools from the OpenCV library (https://opencv.org/ (accessed on 26 November 2025). Considering the observation conditions, combining thresholding analysis and brightness normalization should enable consistent segmentation of features such as:

- The laser spot (determined as the brightest region),
- Surrounding sparking regions,
- High-temperature zones,
- Laser traces,
- The entire exposure area.

Additional bright segments adjacent to the laser spot can be identified as separate regions.

A set of identifying features of the regions of interest in the recorded images of the melt pool was defined, optimized by importance and quantity to ensure sufficiency for monitoring tasks. The feature set includes the collection of triplets {(Si, Xi, Yi)i} normalized relative to the entire region of interest (ROI), where Si is the contour area of the i-th ROI, and Xi, Yi are the coordinates of its center of gravity. Moment characteristics of the contours were additionally analyzed. Importance was determined during classification and regression tasks, including predicting the focal length using random forest-based algorithms. In model training, the out-of-bag error was calculated for each feature element’s subset (out-of-bag error) and averaged across the entire random forest [85]. It was established that a set of 9 features, corresponding to the top three brightness-ranked regions (laser spot, sparking region around the laser spot, high-temperature area), meets the information importance criterion (maximum MSE on out-of-bag feature samples). Additional dimensionality reduction using UMAP to 8 features and 3 synthetic features revealed a significant decrease.

An adaptive noise-robustness algorithm was proposed: using a moving average with an optimal window size of 20–25 frames for feature calculation. Additionally, outlier filtering based on deviations exceeding 2 standard deviations from the moving average of the brightness of the region of interest is possible.

**Figure 16:**
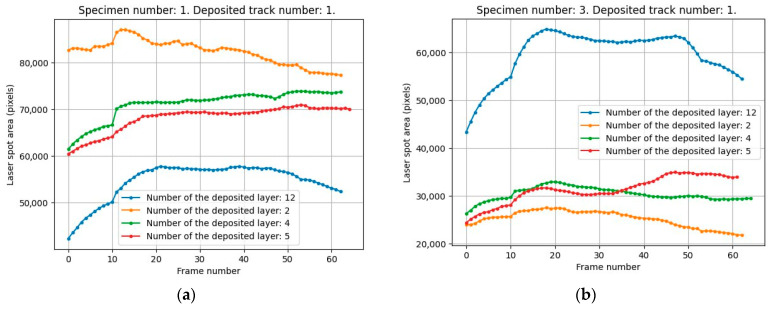
Laser spot area (in pixels) as a function of powder feed rate and focus distance: (**a**)—20% below nominal values, (**b**)—20% above nominal values.

Based on the study results, it can be concluded that the laser spot area detected by the camera (the brightest cluster in the segmented image) exhibits good correlation with both the focus distance and the powder feed rate in the DED process. This fact is confirmed by the data presented in Figure 16. When the powder feed rate and focus distance are reduced by 20% relative to their nominal values, and the height of the built-up specimen increases (i.e., the layer number increases), the laser spot size decreases (Figure 16a). Conversely, when these parameters are increased by 20% (Figure 16b), the laser spot size also increases. This suggests that, since the layer number being deposited can be determined with sufficient accuracy (the average accuracy for the entire dataset is 0.973), deviations of the focus distance from its nominal value—caused by accumulated errors due to external deposition conditions and discrepancies between the theoretical calculated powder consumption and the actual required amount—can be compensated by adjusting the powder feed rate.

Notably, cross-validation revealed the most process-sensitive external factor, which is especially important under complex visual effects inherent in DED. This factor was identified as the layer number being deposited.

It should also be noted that proper focusing, in conjunction with other process parameters maintained at optimal levels, is a necessary condition for forming the desired thermal field within the melt pool to ensure the quality of the DED process. Larger deviations from the nominal parameters tend to destabilize the process—this is reflected in increased prediction errors. This is clearly illustrated by the data in Figure 8 and Figure 9, which show the dependence of *MAE* on the specific melting process parameters (specimen numbers). The ninth process mode exhibits the greatest deviations from the nominal parameters and, correspondingly, the highest *MAE* values.

**Figure 17:**
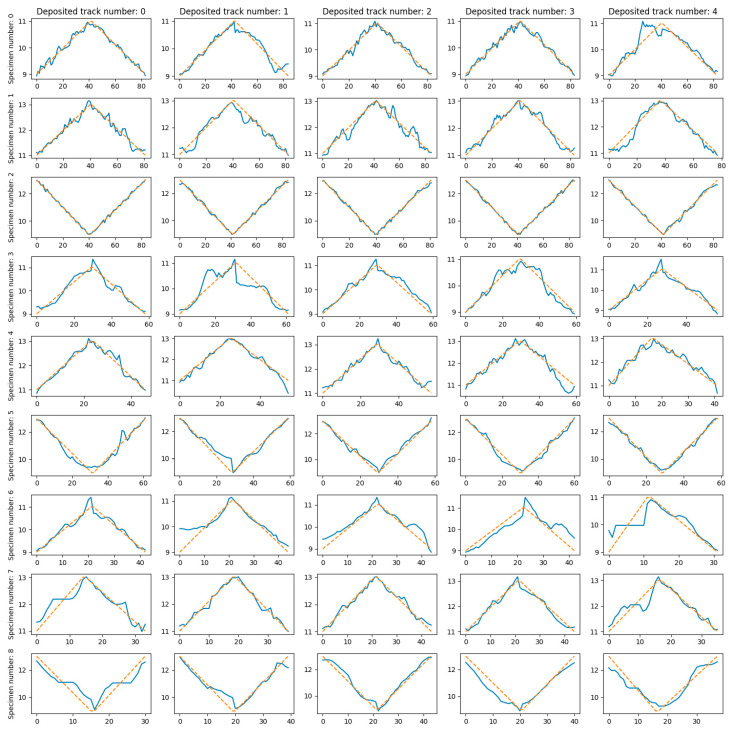
Predicted (solid lines) and labeled (dashed lines) focus distance values.

The curves of predicted and observed focus distance shown in Figure 17 display some outliers in the prediction curves, but a general trend of agreement between these curves is observed. Individual outliers and deviations can be easily corrected in in situ monitoring systems using PID regulators and threshold filters based on deviations.

The main task of the closed-loop system is to monitor and ensure the conditions for Directed Energy Deposition (DED) within the “stability window” (Figure 1). It is assumed that prior testing has been conducted, and the process parameters suitable for this region have been identified. Research shows that, by layers 5–7, the DED process and the molten pool parameters stabilize. At this point, deviations from the optimal build-up conditions are typically not observed. Consequently, the recorded parameters for the melt pool state (Table 7) can be treated as target values, and control can be actively maintained by adjusting the scanning speed and powder feed rate, as well as fine-tuning the focal distance. The proposed method does not require instantaneous reactions; instead, it diagnoses deviations from nominal melting conditions before defects appear. These deviations occur gradually due to changes in the thermal regime during layer-by-layer build-up. Previous studies indicate that the interlayer wait time should be at least 40 s. Otherwise, defects may form in the material due to accumulated thermal energy. Therefore, a system response time of approximately 40 s is sufficient for corrective actions. Image acquisition and a 23-frame moving average consume minimal time: at each moment, one frame is analyzed, and features are averaged with the 22 previously processed frames. This ensures that computational time is significantly less than the 40 s required for process stabilization. Delays only occur during the initial frames processing; afterward, the main task is to process one frame at a time, given that regression and classification operations are computationally lightweight.

The accuracy and sensitivity of the method were evaluated during the fusion of layers 4 and 5 across all specimens, where the focal distance was varied linearly within a range of delta = 2–4 mm (Table 4). The regression model was trained to determine the current focal distance. The achieved training accuracy with the optimal set of identifying features (Table 3) yielded a *MAE* of 0.16488 mm. With a minimal data acquisition frequency of f = 0.5 Hz, the conditional error in the calculated focal distance for each 1 mm increase in the height of the workpiece will be inversely proportional to the number of measurements, which can be expressed as:

$$\begin{array}{l} Error=\;MAE\cdot \frac{delta}{layer\;thickness\cdot interlayer\;wait\;time\;\cdot f}=\\ =0.16488\cdot \frac{\left(2\ldots 4\right)}{0.8\cdot 40\cdot 0.5}\approx 0.02\ldots 0.04\;\mathrm{mm}. \end{array}$$

This accuracy is acceptable for adjusting the focal distance, for example, after every 10 layers.

## 5. Conclusions

1. A monitoring method for DED processes was proposed and tested, based on the following principles:
  - To prevent the occurrence of characteristic defects, it is sufficient to monitor key technological parameters of DED—laser power, scanning speed, and powder feed rate—in the process stability region.
  - During a stable (defect-free) DED process, the molten pool is characterized by specific temperature distribution areas, which can be registered by an embedded optical camera and segmented into clusters based on normalized brightness using the proposed segmentation and machine learning methods.
  - External conditions changes influence the identified characteristics of these clusters observed during the study.
  - By controlling the variability of these features within a closed-loop monitoring system through key DED process parameters, it is possible to maintain the process within its stable operating region.
2. The best classification results for the number of deposited layers, scanning speed, and powder feed rate based on the identification features were achieved using the RandomForestClassifier and HistGradientBoostingClassifier, highlighting the effectiveness of ensemble methods for this problem.
3. An effective focus distance estimation algorithm (regressor) was developed, based on the same principles as those used in classification tasks. For regression, models such as HistGradientBoostingRegressor and ExtraTreesRegressor were used. The latter demonstrated superior accuracy based on the chosen metric.
4. The recorded images of the melt pool obtained from the camera enable machine learning methods to determine the laser spot area with high precision (above 0.99). This area, as modeled by regression, is consistently linked to the focal length, powder feed rate, and layer number. Adjusting the powder flow can compensate for the variability in DED conditions affecting the spot size and ensure proper focusing, which is vital for forming high-quality layers during laser deposition. It should be understood that, for a different material, a regression model may require retraining.

## Figures and Tables

**Table 1:** Percentage composition of chemical elements in Cr32Ni50W4.5Mo2.5TiAl powder.

Element	Powder
Ni	Balance
C	≤0.10
S	≤0.010
P	≤0.015
Mn	≤0.50
Cr	32.0–35.0
W	4.30–5.30
Ti	0.50–1.10
Si	≤0.40
Nb	0.50–1.10
Mo	2.30–3.30
Fe	≤4.00
Al	0.50–1.10
Ce	≤0.030
B	≤0.008

**Table 2:** Orthogonal L9:4 Taguchi plan for 3 factors.

No	Scanning Speed V, mm/s	Actual Focus Distance f, mm	Powder Feed Rate for Internal Tracks, C g/min
0 **	25	11	25.6
1	20	9	20.5
2	20	11	25.6
3	20	13	30.7
4	25	9	25.6
5	25	11	30.7
6	25	13	20.5
7	30	9	30.7
8	30	11	20.5
9	30	13	25.6

** Nominal technological mode.

**Table 3:** Unchangeable technological parameters.

Parameter	Value
Laser power, P, W	2000
Single track width, h, mm	2.5
Layer height, t, mm	0.8
Overlap ratio between tracks, O, %	67
Process pause duration, t_p, s	40

**Table 4:** Plan of the image registration process of the molten pool for creating the DataSet.

No	Layer	Note
All the technological parameters of deposition should be taken from the experimental plan of Table 2 and Table 3		
1	2	
2	7	
3	12	
4	4 (for specimens No 1, 4, 7)	the focusing distance in the layer is changed linearly from 9 mm (start of the track) to 11 mm (end of the track).
5	5 (for specimens No 1, 4, 7)	the focusing distance in the layer is changed linearly from 11 mm (start of the track) to 9 mm (end of the track).
6	4 (for specimens No 2, 5, 8)	the focusing distance in the layer is changed linearly from 11 mm (start of the track) to 13 mm (end of the track).
7	5 (for specimens No 2, 5, 8)	the focusing distance in the layer is changed linearly from 13 mm (start of the track) to 11 mm (end of the track).
8	4 (for specimens No 3, 6, 9)	the focusing distance in the layer is changed linearly from 13 mm (start of the track) to 9 mm (end of the track).
9	5 (for specimens No 3, 6, 9)	the focusing distance in the layer is changed linearly from 9 mm (start of the track) to 13 mm (end of the track).

**Table 5:** Description of features and their identifiers in CSV files.

$\mathrm{Feature}\;x_{k}$ Name	$\mathrm{Feature}\;x_{k}$ Identifier	Dimension
Laser spot area (red area)	S0	pixel^2^
The area of the sparking region around the laser spot (blue area)	S1	pixel^2^
High-temperature region area (purple area)	S2	pixel^2^
Coordinates of the center of gravity of the laser spot relative to the exposure center	X0, Y0	pixel
Coordinates of the center of gravity of the sparking region relative to the exposure center	X1, Y1	pixel
Coordinates of the center of gravity of the high-temperature region relative to the exposure center	X2, Y2	pixel

**Table 6:** Dataset fragment with a description of the features.

	$S_{0}$	$X_{0}$	$Y_{0}$	$S_{1}$	$X_{1}$	$Y_{1}$	$S_{2}$	$X_{2}$	$Y_{2}$
**count**	6665	6665	6665	6665	6665	6665	6665	6665	6665
**mean**	0.213	0.001	0.209	0.254	−0.003	0.204	0.309	−0.005	0.203
**std**	0.079	0.024	0.024	0.100	0.021	0.021	0.137	0.022	0.022
**min**	0.099	−0.049	0.159	0.126	−0.052	0.156	0.143	−0.047	0.160
**25%**	0.134	−0.021	0.187	0.156	−0.023	0.185	0.179	−0.022	0.186
**50%**	0.207	0.000	0.207	0.232	−0.003	0.204	0.262	−0.007	0.200
**75%**	0.279	0.021	0.229	0.329	0.012	0.220	0.416	0.011	0.219
**max**	0.378	0.051	0.258	0.488	0.037	0.245	0.646	0.053	0.260

**Table 7:** Minimum error achieved depending on the number of features.

$S_{0}$	$X_{0}$	$Y_{0}$	$S_{1}$	$X_{1}$	$Y_{1}$	$S_{2}$	$X_{2}$	$Y_{2}$	*MAE*
	x								0.7639
	x					x			0.5598
			x	x			x		0.4425
			x	x		x	x		0.3121
	x		x	x		x	x		0.2466
x	x		x	x		x	x		0.1648
x	x		x	x		x	x	x	0.1637
x	x	x	x	x		x	x	x	0.1633
x	x	x	x	x	x	x	x	x	0.1629

The highlighted row corresponds to the best features combination.

## Data Availability

The data presented in this study are available on request from the corresponding author.

## Abbreviations

The following abbreviations are used in this manuscript:

DED	Laser Directed Energy Deposition
IR	Infrared radiation
DED	Directed Energy Deposition
L-PBF	Laser Powder Bed Fusion
LoF	Lack of fusion
ML	Machine learning
KNN	K-Nearest Neighbour
MPCA	Multilinear principal component analysis
SVM	Support vector machine
SVD	Singular value decomposition
GMM	Gaussian mixture model
DL	Deep learning
GAN	Generative adversarial network
2D	2-dimensional
HAZ	Heat-Affected Zone
TP	True positive
TN	True negative
FP	False positive
FN	False negative
*MAE*	Mean Absolute Error
UMAP	Uniform Manifold Approximation and Projection
ETR	Extra-Trees Regressor
GPR	Gaussian Process Regression
ROI	Region of interest
PID	Proportional–Integral–Derivative
DBI	Davies–Bouldin index
